# Acute renal failure associated with nonfulminant acute viral hepatitis A

**DOI:** 10.4103/0971-4065.42344

**Published:** 2008-04

**Authors:** S. Sarawgi, A. K. Gupta, D. S. Arora, S. Jasuja

**Affiliations:** Department of Internal Medicine, Indraprastha Apollo Hospitals, Sarita Vihar, Mathura Road, New Delhi - 110 044, India; 1 Department of Nephrology, Indraprastha Apollo Hospitals, Sarita Vihar, Mathura Road, New Delhi - 110 044, India; 2 Department of Histopathology, Indraprastha Apollo Hospitals, Sarita Vihar, Mathura Road, New Delhi - 110 044, India

**Keywords:** Acute interstitial nephritis, acute renal failure, myocarditis, nonfulminant hepatitis A, pancreatitis

## Abstract

Hepatitis A runs a benign course in children, but may have atypical presentations in adults. Very rarely acute renal failure complicates nonfulminant hepatitis A. We report a patient with nonfulminant acute viral hepatitis A with multiorgan involvement. Patient had biopsy proven acute interstitial nephritis, acute pancreatitis, acute myocarditis and required hemodialysis for 6 weeks.

Hepatitis A is usually a mild self-limiting infection of liver. Acute renal failure (ARF) complicating acute nonfulminant viral hepatitis A is exceedingly rare although it is not an uncommon association in cases of fulminant hepatitis with massive hepatic necrosis due to hepatitis A.^1^

We are presenting here a case of a 25-year-old young male who developed ARF, acute pancreatitis, and myocarditis in the course of viral hepatitis A. The patient had prolonged ARF requiring dialysis.

## Case Report

A 25-year-old young male, a resident of Assam, presented with history of fever, yellowish discoloration of sclera and urine, poor appetite, vomiting, pain upper abdomen, and decreased urine output for 1 week. He got admitted at a local hospital. Hospital summary showed that he was hemodynamically stable, had hepatomegaly, mild leucocytosis, deranged liver functions (AST 4404 U/L; ALT 4692 U/L; total/direct bilirubin 9.8/5.3 mg/dl; prothrombin time (PT) (patient/control) 31/13 s; and deranged renal parameters (urea 154 mg/dl; creatinine 10.3 mg/dl; sodium 121 mg/dl; potassium 3.2 mg/dl). He was initiated on hemodialysis. After 1 week, his liver functions started improving; however, he remained oligoanuric and uremic, and was subsequently referred to our hospital. At presentation, he had a BP of 170/90 mm Hg, tachycardia, tachypnoea, and low oxygen saturation. Examination revealed moderate pallor, icterus, raised jugular venous pressure (JVP), anasarca, auscultation showed bilateral crackles, loud S1 and normal S2, audible third heart sound, hepatomegaly, epigastric tenderness, and ascites. Laboratory investigations revealed Hb 7.5 g/dl; TLC 14,600 cells/mm^3^; DLC N-79/L-14/M-04%, ESR 140 mm/first hour; amylase 243 IU/L; lipase 1565 IU/L; AST 92 IU/L; ALT 111 IU/L; ALP 826 IU/L; SGGPT 436 IU/L; bilirubin (total/direct) 6.2/3.2 mg/dl; urea 114 mg/dl; creatinine 10.4 mg/dl; PT (patient/control) 11.6/11 s: sodium 135 meq/l; potassium 4.0 meq/l. Anti-HAV IgM was positive and rest of the serologies were negative for other viruses, malaria, and leptospira. Urine R/M examination showed albumin++, pus cells: 4-6/hpf, RBC: 25-30/hpf; 24 h urine protein - 1 g/day; blood and urine culture did not show any growth; ultrasound showed enlarged liver, increased echogenicity of kidneys, ascites, and bilateral pleural effusions. Electrocardiogram (ECG) showed sinus tachycardia. Echo Doppler of heart showed features of myocarditis with low ejection fraction (40%), global hypokinesia, and raised left ventricular end diastolic pressure. C3, C4, ANA, and ANCA were negative. Serum lactate dehydrogenase (LDH) and creatine phosphokinase (CPK) were normal with no fragmented red blood cells (RBCs) in peripheral smear. Noncontrast computerised tomographic (CT) scan excluded extensive pancreatitis. A provisional diagnosis of acute viral hepatitis A with ARF, toxic myocarditis, and acute pancreatitis was made. The patient was continued on hemodialysis and other supportive treatment.

**Fig. 1:**
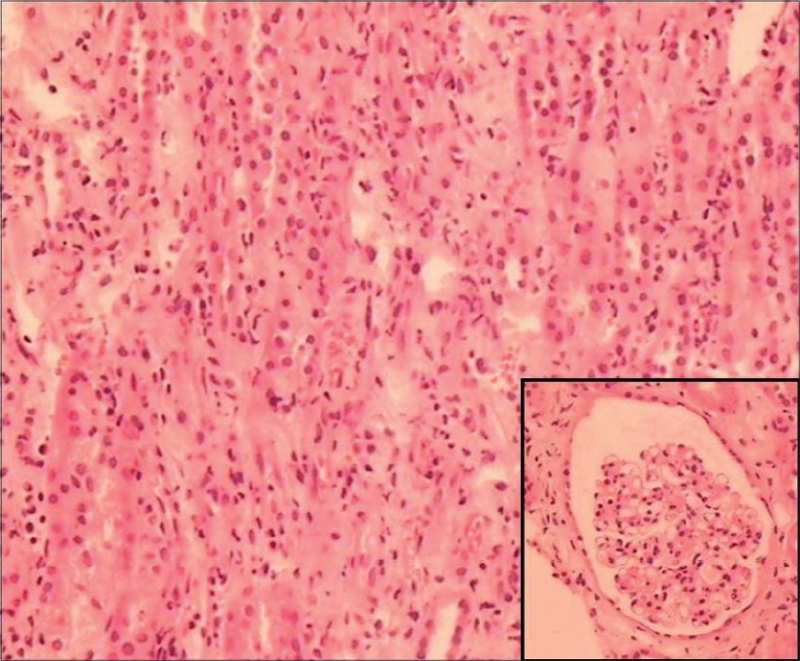
Moderate mixed inflammatory cell infiltrate in the interstitium, (H&E, ×4). Inset: unremarkable glomerulus

**Fig. 2:**
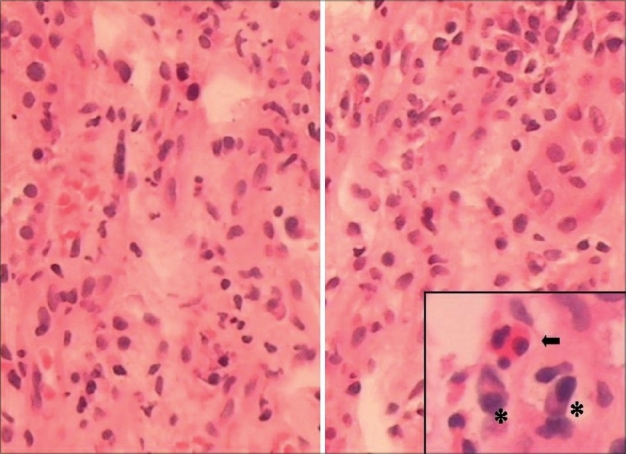
Interstitial infiltrate consists of lymphocytes, neutrophils, plasma cells (*), and eosinophils (*arrow*), (H&E, ×20)

For the next 5 weeks, he remained stable with gradually improving liver, cardiac, and pancreatic functions, but remained anuric requiring dialysis. Kidney biopsy was done after 6 weeks of onset of illness to know exact nature of renal involvement and for future prognostication. Light microscopy [Figs. 1 and 2] showed normal glomeruli, patchy moderate mixed inflammatory cell interstitial infiltrate composed of lymphocytes, plasma cells, neutrophils, and a few eosinophils. Mild interstitial fibrosis was also present. The tubular epithelium showed flattening and desquamation in focal areas. Blood vessels appeared normal. Immunofluorescence was negative for IgA, IgG, IgM, C3, C1q, and fibrinogen.

After about 6 weeks of anuria and hemodialysis, his urine output gradually increased. Renal parameters also declined slowly and thus dialysis was withheld. After about 2 weeks further his investigations showed Hb 8.6 mg/dl; TLC 8,700 cells/mm^3^; bilirubin (total/direct) 0.7/0.2 mg/dl; urea 30 mg/dl; creatinine 1.2 mg/dl; AST 48 IU/L; ALT 20 IU/L; and lipase 73 IU/L.

Repeat urine examination, ECG and echocardiography were normal.

## Discussion

Typical presentations of viral hepatitis A include an asymptomatic form seen in young children under the age of 5 years and symptomatic form seen in relatively older children and adults, characterized by malaise, fatigue, and icteric hepatitis.^2^ Very rarely nonfulminant hepatitis A has lead to ARF.^3^–^5^ When such a patient develops renal failure, common causes such as sepsis, shock, drug toxicity, bacterial infections, and rhabdomyolysis should be ruled out first. Although hepatitis A is usually a benign self-limited disease; however, in the present case it was associated with three unusual manifestations namely acute interstitial nephritis leading to prolonged oliguric ARF requiring dialysis, acute pancreatitis, and myocarditis. Patient had only acute hepatitis A infection as is confirmed by reactive hepatitis A IgM antibody. He required multiple sessions of hemodialysis for 6 weeks and gradually recovered normal hepatic and renal functions with normalization of pancreatic enzymes and myocarditis. The prognosis of the renal failure due to hepatitis A virus (HAV) infection is good, although the recovery was substantially delayed. Pancreatitis was diagnosed on the basis of characteristic epigastric pain radiating to back, a small sympathetic effusion, and very high pancreatic enzymes, which improved along with improvement of renal and liver functions. Myocarditis was also self-limiting, suspected on clinical grounds, and confirmed by Echo-Doppler.

Shroff *et al.* reported two cases of ARF associated with hepatitis A out of which one patient required dialysis. They suggested beneficial role of high dose acetylcysteine in recovery of renal function.^3^ Vesely *et al.* related the peak bilirubin levels to the requirement of hemodialysis in patients who developed ARF in the setting of viral nephritis.^5^ There also seems to be temporal relationship between improvement of the bilirubin concentration and improvement in renal functions.^5^,^6^

Faust and Pimstone reviewed the literature on ARF-associated with hepatitis A infection. Acute tubular necrosis (ATN) was the most common histopathological diagnosis. Acute interstitial nephritis, mesangial proliferative glomerulonephritis, and normal histology were also reported as single cases.^7^ Vaboe *et al.* described combination of interstitial nephritis and ATN associated nonfulminant hepatitis A requiring dialysis support.^8^ Also viral hepatitis A has been rarely associated with cardiac manifestations^9^ and pancreatitis.^10^ We could not find any such case with combination of renal, cardiac, and pancreatic involvement associated with viral hepatitis A in the literature.

Several mechanisms have been postulated in the pathogenesis of ATN in such settings; these include insufficient renal blood flow due to developing endotoxemia or due to direct cytopathic effect of the virus; while mesangial profilerative glomerulonephritis and interstitial nephritis are due to immune complexes or cryoglubulinemia.

Present case demonstrates that hepatitis A infection can be very severe, resulting in acute interstitial nephritis requiring prolonged dialysis support, myocarditis, and pancreatitis in an apparently healthy individual. Thus in the course of nonfulminant hepatitis A, renal functions should be closely monitored.
